# Seroprevalence and risk factors of caprine brucellosis in Khartoum state, Sudan

**DOI:** 10.14202/vetworld.2018.511-518

**Published:** 2018-04-19

**Authors:** Eman Mohamed-Ahmed Mohamed, Abdelhamid Ahemd Mohamed Elfadil, Enaam Mohamed El-Sanousi, Hatim Hamad Ibrahaem, Saad El-Tiab Mohamed-Noor, Mohamed Abdelsalam Abdalla, Yassir Adam Shuaib

**Affiliations:** 1 Department of Veterinary Preventive Medicine, College of Veterinary Medicine, Sudan University of Science and Technology, P.O. Box 204 (Hilat Kuku), Khartoum North, Sudan; 2 Department of Brucellosis, Veterinary Research Institute, Soba, P.O. Box 8067, Khartoum, Sudan

**Keywords:** brucellosis, goats, risk factors, rose bengal plate test, seroprevalence, Sudan

## Abstract

**Aim:**

This cross-sectional study was conducted from April to July 2012 in Khartoum state, Sudan, to determine the seroprevalence of brucellosis in goats and to investigate potential risk factors associated with this disease.

**Materials and Methods:**

A total of 307 serum samples were collected from both sexes of goats in four different localities and were subjected to testing for brucellosis using rose bengal plate test (RBPT), serum agglutination test (SAT), and competitive enzyme-linked immunosorbent assay (cELISA).

**Results:**

The overall seroprevalence was 11.4% (n=35) with a 95% confidence interval (CI) ranging from 7.80 to 15.0. Out of these 35 RBPT-positive samples, the positivity of 18 and 17 were confirmed by SAT and cELISA, respectively. A significant statistical variation was observed between brucellosis seroprevalences in goats purchased from local animal markets and goats that were raised at the farm. Conversely, such significant variations were not observed among the categories of other risk factors with seroprevalences ranging from 3.0% (95% CI between 0.40 and 7.20) to 16.3% (95% CI between 10.4 and 22.3). Location (*χ*^2^=9.33, df=3, p=0.02), breed (*χ*^2^=3.52, df=1, p=0.05), herd size (*χ*^2^=6.59, df=2, p=0.03), and herd expansion (*χ*^2^=5.39, df=1, p=0.02) were associated with RBPT-positive status for brucella in the two-tailed Chi-square test. In addition, Sharq an-Nil locality and goats raised at the farm had increased odds of being RBPT positive.

**Conclusion:**

Brucellosis was detected in goats in all surveyed localities. An effort should be made to educate goat owners/herders about brucellosis as well as about the importance of vaccination.

## Introduction

Brucellosis is an infectious bacterial disease of domestic and wild animals with a serious zoonotic implication [1,2]. This disease is a significant public health problem in many parts of the world [1]. Brucellosis is caused by bacteria from the genus *Brucella* which contains the following species: *Brucella abortus*, *Brucella canis*, *Brucella ceti*, *Brucella inopinata*, *Brucella melitensis*, *Brucella neotomae*, *Brucella ovis*, *Brucella papionis*, *Brucella microti*, *Brucella pinnipedialis*, *Brucella suis*,and *Brucella vulpis*. All these bacteria are small, non-motile, aerobic, facultative intracellular, and Gram-negative coccobacilli [2]. *B. melitensis* is the most pathogenic species for humans in comparison with other brucellae, and primary reservoirs of this *Brucella* species are small ruminants [3]. Socio-economic factors play a major role in spreading of *B. melitensis*. In Africa, it is endemic and results in considerable economic losses due to abortion, fertility problems, and less milk yield [3-5]. In addition, *B. melitensis* is one of the significant barriers of trade of small ruminants [4-7].

Sudan has the biggest goat population in East Africa which is estimated to be around 43 million head [8,9]. The vast majority of these goats are indigenous ecotypes such as Nubian, desert, Nilotic, or mountain goats. The remaining minority are foreign breeds such as Shami and Saanen or their crosses with indigenous ecotypes [10]. From an economic point of view, goats play an important role in the national economy of Sudan. In addition, more than half of the Sudanese households own goats for milk and financial reasons [9]. The prevalence of infectious diseases, such as brucellosis, is one of the major constraints that hinder the development of the national population of goats in Sudan [11].

The prevalence of brucellosis among animals in sub-Saharan Africa is poorly estimated or unknown in some cases. In addition, because of the economic status of most of the African countries, control of brucellosis has been very difficult [5]. Knowledge of risk factors that enhance spreading of infectious diseases is an important prerequisite for effective control, management, and eradication of these diseases [7,12]. Many factors influence the susceptibility of animals to brucellosis including natural resistance, age, sex, breed, level of immunity, and environmental stress [6,7]. Despite the presence of large population of goats in Sudan, a few numbers of studies addressed brucellosis in goats. Most of these studies were serosurveys [11,13,14]. However, only a few, if any at all, did include investigations on potential risk factors contributing to the occurrence and spreading of brucellosis amid goat populations. Therefore, the objectives of this study were to determine the seroprevalence of brucellosis in goats in Khartoum state and to investigate the potential risk factors associated with this disease.

## Materials and Methods

### Ethical approval

Ethical approval is not necessary for such type of study.

### Informed consent

Informed consent has been obtained from the participants.

### Study area

This study was conducted in Khartoum state, latitudes 15° and 16° North and longitudes 31° and 34° East. The total area of the state is 20,736 km^2^. It falls within the desert and semi-desert ecological zones of Sudan. The state has a hot to very hot climate with rainfall ranging from 100 to 300 mm during summer (25-40°C) and warm to cold, dry weather in winter (15-25°C). Khartoum state is divided into seven localities namely, Karari, Om Badda, Omdurman, Bahri, Sharq an-Nil, Khartoum, and Jabal Awliya. Each locality is subdivided into smaller administrative units (between 4 and 10). The total numbers of livestock in Khartoum state were estimated to be around 1,384,000 heads of animals from which 240,000 (17%) are cattle, 513,000 (37%) are sheep, 624,000 (45%) are goats, and nearly 7,000 (1%) are camels [8].

### Study population

The study population included both sexes of goats as well as various age groups and breeds that are either kept in farms or houses in Khartoum state. The number of goats in each of the localities of Khartoum state is as follows: 319,377 (51%) in Sharq an-Nil, 100,873 (16.1%) in Bahari, 92,242 (14.7%) in Omdurman, 46,710 (7.4%) in Karari, 37,578 (6%) in Om Badda, 17,819 (2.8%) in Jebal Awlia, and 9,562 (1.5%) in Khartoum [8]. In general, semi-intensive production system predominates for goats raising in Khartoum.

### Study design and sample size

A cross-sectional study with a multistage sampling strategy was carried out between April and July 2012 [15]. Out of the seven localities of the state, four were randomly selected. In each selected locality, administrative units, flocks, and individual animals were conveniently and/or randomly selected. The number of sampled administrative units was proportional to the total number of administrative units in the locality [15].

The sample size (n) was determined using the standard formula of Thrusfield [15]. A 95% confidence interval (CI) and absolute precision of 5% were selected for calculating n, which was after that determined to be 307 animals.

### Serum samples collection

A volume of 3-5 mL of venous blood was collected from the jugular vein of each selected animal using clean, sterilized, and labeled plain vacutainer tubes and needles [16]. The collected blood samples were allowed to clot and transported on ice to the Laboratory of Microbiology, College of Veterinary Medicine, Sudan University of Science and Technology, Khartoum North, Sudan. In the laboratory, the samples were centrifuged at 1000 rpm for 5 min for proper separation of the serum which was decanted into clean, sterile screwcapped Eppendorf tubes and frozen at −20°C until used.

### Diagnostic tests

The collected samples were tested at the Department of Brucellosis, Veterinary Research Institute, Soba, Khartoum, Sudan. All samples were first screened using rose bengal plate test (RBPT). Subsequently, the positivity and antibodies titers of RBPT-positive samples were confirmed and measured using serum agglutination test (SAT) and competitive enzyme-linked immunosorbent assay (cELISA),respectively.

The RBPT and the SAT were carried out as described by OIE [16] while the cELISA was conducted according to the instructions of the manufacturer. The cELISA kit was obtained from the Veterinary Laboratories Agency, Addlestone, UK.

### Questionnaire survey

A semistructured questionnaire was designed to mine data about potential risk factors that enhance the spreading of brucellosis in goats. The questionnaires were administered and discussed with owners/herders of goats based on willingness. The investigated factors were selected after reviewing related published literature. The questionnaire had closed-ended type questions that were classified into categories: flock characteristics such as flock size and flock management such as sources of replacement animals, purpose of production, and so on. Individual animal details such as sex, age, history of abortion, and retained placenta were recorded.

### Statistical analysis

Statistical analyses were carried out using the Statistical Package for Social Sciences (SPSS) for Windows^®^ version 22.0 (SPSS Inc., Chicago, Illinois). First, descriptive statistics, frequencies, and cross-tabbing were computed. Second, univariate and multivariate analyses using the two-tailed Chi-square test and logistic regression model were conducted. Associations in the Chi-square test and logistic regression model were deemed significant when p≤0.05. All potential risk factors with a p≤0.25 in the chi-square test were entered into the final logistic regression model.

## Results

### Overall prevalence and confirmed positivity

The overall seroprevalence of anti-brucella antibodies in goats using RBPT was 11.4% (n=35) with a 95% CI ranging from 7.80 to 15.0. Out of these 35 RBPT-positive samples, the positivity of 18 and 17, respectively, were confirmed using SAT, with antibody titers of equal or more than 50 IU/mL and cELISA.

### Seroprevalences of anti-brucella antibodies by risk factors

The difference between the seroprevalences of anti-brucella antibodies in goats purchased from local animal markets (2.0%, n=1, 95% CI from 0.10 to 5.80) and goats that were raised in the farm (13.3%, n=34, 95% CI from 9.10 to 17.4) for the purpose of replacement of culled goats and/or flock expansion was statistically significant. However, such significant statistical differences were not observed among the categories of all other investigated individual animal and management risk factors (Tables-1 and 2).

**Table-1:** Estimated seroprevalences of brucellosis in goats in Khartoum state (April-July 2012).

Risk factors	Number of tested	Number of positive	Percentage	95% CI lower-upper
Localities				
Sharq an-Nil	147	24	16.3^a^	10.4-22.3
Om Badda	19	3	15.8^a^	3.40-39.6
Omdurman	75	6	8.00^a^	1.90-14.1
Jabal Awliya	66	2	3.00^a^	0.40-7.20
Breeds				
Local	95	6	6.30^a^	1.40-11.2
Cross	212	29	13.7^a^	9.11-18.3
Age				
≤ 1 year	67	6	9.00^a^	2.10-15.8
> 1 year	240	29	12.1^a^	8.00-16.2
Sex				
Male	23	2	8.70^a^	1.10-20.2
Female	284	33	11.6^a^	7.90-15.3
Abortion				
No	138	12	8.70^a^	4.00-13.4
Yes	146	21	14.4^a^	8.70-20.1
History of RP				
No	191	19	9.90^a^	5.70-14.2
Yes	93	14	15.1^a^	7.79-22.3
Total	307	35	11.4	7.80-15.0

Different superscripts indicate significant difference. No=Number, CI=Confidence interval, RP=Retained placenta, and *=Constant category (ies)

**Table-2:** Estimated seroprevalences of brucellosis in goats in Khartoum state (April-July 2012).

Risk factors	Number of tested	Number positive	Percentage	95% CI lower-upper
Herd size				
≤10 Animals	68	2	2.90^a^	0.40-10.3
1050 Animals	141	21	14.9^a^	9.00-20.8
>50 Animals	98	12	12.2^a^	5.90-18.7
P of P				
Meat	0	0	0.00*	0.00-00.0
Milk	181	19	10.5^a^	6.00-20.8
Mixed	126	16	12.7^a^	6.90-18.5
Veterinary services				
Inaccessible	192	26	13.5^a^	8.70-18.4
Accessible	115	9	7.80^a^	2.90-12.7
Grazing				
No	187	22	11.8^a^	7.10-15.5
Yes	120	13	10.8^a^	5.30-16.4
fFE and/or AR				
Purchased	51	1	2.00^a^	0.10-5.80
Raised in farm	256	34	13.3^b^	9.10-17.4
Water source				
Common canal	76	10	13.2^a^	5.60-20.8
Well	130	17	13.1^a^	7.30-18.9
Tap water	101	8	7.90^a^	2.70-13.2
PS				
Extensive	131	15	11.5^a^	6.00-16.9
Intensive	176	20	11.4^a^	6.70-16.1
Feed source				
Farm	30	5	16.7^a^	3.30-30.0
Market	242	30	12.4^a^	8.30-16.5
Food remain	0	0	0.00*	0.00-0.00
Mixed	35	0	0.00*	0.00-0.00
SOM for breeding				
No	190	21	11.1^a^	6.60-15.5
Yes	117	14	12.0^a^	6.10-17.8
D of FM				
No	14	3	21.4^a^	4.70-42.9
Yes	293	32	10.9^a^	7.40-14.5
Vaccination				
No	307	35	11.4^a^	7.80-15.0
Yes	0	0	0.00*	0.00-0.00
Parturition pen				
No	307	35	11.4^a^	7.80-15.0
Yes	0	0	0.00*	0.00-0.00
C with other As				
No	89	10	11.2^a^	4.80-17.8
Yes	218	25	11.5^a^	7.20-15.7
Stray dogs				
No	148	14	9.50^a^	4.70-14.2
Yes	159	21	13.2^a^	7.90-18.5

Different superscripts indicate significant difference. No=Number, CI=Confidence interval, P of P=Purpose of production, FE and/or AR=Flock expansion and/or animal replacement, PS=Production system, SOM for breeding=Share one male for breeding between farms/flocks, D of FM=Disposal of fetal membranes, C with other As=Contact with other animals,

* = constant category (ies)

The seroprevalences ranged from 3.0% (95% CI between 0.40 and 7.20) to 16.3% (95% CI between 10.4 and 22.3) for the categories of the investigated individual animal risk factors (Table-1). While for the categories of the investigated management risk factors, seroprevalences ranged from 0.0% (95% CI from 0.00 to 0.00) to 21.4% (95% CI from 4.70 to 42.9). Moreover, four management risk factors, namely, purpose of production (for what reason goats are being raised), source of animal feed, whether goats were vaccinated for protection against brucella or not, and whether there is a separate pen for kidding or not were risk factors with constant category(ies), i.e., category with 0 number of investigated samples (Tables-1 and 2).

### Association of risk factors with RBPT positivity

There were differences between the proportions of seropositive samples among the investigated categories of the individual animal and management risk factors. Two individual animal and two management risk factors were associated with the seropositive status of RBPT at p≤0.05 (Tables-3 and 4). These were location (*χ*^2^=9.33, df=3, p=0.02), breed (*χ*^2^=3.52, df=1, p=0.05), herd size (*χ*^2^=6.59, df=2, p=0.03), and herd expansion (*χ*^2^=5.39, df=1, p=0.02). However, the remaining individual animal and management risk factors were not associated with the RBPT positivity in the two-tailed Chi-square test (Tables-3 and 4).

**Table-3:** Univariate association between brucellosis positivity and potential risk factors in goats in Khartoum state (April-July 2012).

Risk factors	Number tested (%)	df	Χ^2^	p value
Localities				
Sharq an-Nil	147 (16.3)	3	9.33	0.03
Om Badda	19 (15.8)			
Omdurman	75 (8.00)			
Jabal Awliya	66 (3.00)			
Breeds				
Local	95 (6.30)	1	3.52	0.05
Cross	212 (13.7)			
Age				
≤ 1 year	67 (9.00)	1	0.50	0.42
> 1 year	240 (12.1)			
Sex				
Male	23 (8.70)	1	0.18	0.67
Female	284 (11.6)			
Abortion				
No	138 (8.70)	1	2.23	0.13
Yes	146 (14.4)			
History of RP				
No	191 (9.90)	1	1.58	0.22
Yes	93 (15.1)			

No=Number, df=Degree of freedom, RP=Retained placenta

**Table-4:** Univariate association between brucellosis positivity and potential risk factors in goats in Khartoum state (AprilJuly 2012).

Risk factors	Number of tested (%)	df	Χ^2^	p-value
Herd size				
≤10 animals	68 (2.90)	2	6.59	0.04
1050 animals	141 (14.9)			
>50 animals	98 (12.2)			
P of P				
Meat	0 (0.00)	2	-	-
Milk	181 (10.5)			
Mixed	126 (12.7)			
Veterinary services				
Inaccessible	192 (13.5)	1	2.32	0.13
Accessible	115 (7.80)			
Grazing				
No	187 (11.8)	1	0.06	0.80
Yes	120 (10.8)			
FE and/or AR				
Purchased	51 (2.00)	1	5.39	0.02
Raised in farm	256 (13.3)			
Water source				
Common canal	76 (13.2)	2	1.80	0.41
Well	130 (13.1)			
Tap water	101 (7.90)			
PS				
Extensive	131 (11.5)	1	0.001	0.98
Intensive	176 (11.4)			
Feed source				
Farm	30 (16.7)	3	-	-
Market	242 (12.4)			
Food remain	0 (0.00)			
Mixed	35 (0.00)			
SOM for breeding				
No	190 (11.1)	1	0.06	0.81
Yes	117 (12.0)			
D of FM				
No	14 (21.4)	1	1.46	0.23
Yes	293 (10.9)			
Vaccination				
No	307 (11.4)	1	-	-
Yes	0 (0.00)			
Parturition pen				
No	307 (11.4)	1	-	-
Yes	0 (0.00)			
C with other As				
No	89 (11.2)	1	0.03	0.95
Yes	218 (11.5)			
Stray dogs				
No	148 (9.50)	1	1.06	0.30
Yes	159 (13.2)			

No=Number, CI=Confidence interval, P of P=Purpose of production, FE and/or AR=Flock expansion and/or animal replacement, PS=Production system, SOM for breeding=Share one male for breeding between farms/flocks, D of FM=Disposal of fetal membranes, C with other As=Contact with other animals, -=Constant category (ies)

The logistic regression analysis assessed the combined relationship between the potential risk factors that had a p≤0.25 in the univariate analysis. “The regression coefficients (Exp[B]) express “odds ratios” (OR) (= the increased or decreased probability [OR≠1]) of seropositivity occurrence in comparison to the reference (OR=1)” [12]. Sharq an-Nil locality (OR=6.24, 95% CI from 1.45 to 27.6, p=0.015) and goats raised in the farm (OR=7.77, 95% CI between 1.02 and 57.6, p=0.047) were associated with increased odds of being RBPT positive. On the contrary, breeds, history of abortion, history of retained placenta, herd size, accessibility to veterinary services, and disposal of fetal membranes were not associated with RBPT positivity (Table-5).

**Table-5:** Multivariate association of potential risk factors withpositive brucellosis status in goats in Khartoum state (April-July 2012).

Risk factors	Number of tested	Number of positive	Percentage	Exp (B)	95% CI lower-upper	p value
Localities						
Jabal Awliya	66	2	3.00	ref		
Sharq an-Nil	147	24	16.3	6.24	1.4527.6	0.015
Om Badda	19	3	15.8	6.00	0.9239.0	0.061
Omdurman	75	6	8.00	2.78	0.3722.2	0.220
Breeds						
Local	95	6	6.30	ref		
Cross	212	29	13.7	2.53	0.945.87	0.067
Abortion						
No	138	12	8.70	ref		
Yes	146	21	14.4	2.22	0.835.98	0.134
History of RP						
No	191	19	9.90	ref		
Yes	93	14	15.1	1.57	0.763.24	0.221
Herd size						
≤10 Animals	68	2	2.90	ref		
1050 Animals	141	21	14.9	1.51	0.218.36	0.763
>50 Animals	98	12	12.2	1.40	0.228.40	0.756
Veterinary services						
Accessible	115	9	7.80	ref		
Inaccessible	192	26	13.5	2.01	0.834.91	0.132
FE and/or AR						
Purchased	51	1	2.00	ref		
Raised in farm	256	34	13.3	7.66	1.0257.6	0.047
D of FM						
Yes	293	32	10.9	ref		
No	14	3	21.4	2.87	0.558.40	0.238

No=Number, Exp (B)=Regression coefficients, ref=Exp (B) equals 1, RP=Retained place, FE and/or AR=Flock expansion and/or animal replacement, D of FM=Disposal of fetal membranes

## Discussion

The present study was conducted to determine the seroprevalence of brucellosis in goats in Khartoum state and to investigate the potential risk factors associated the seroprevalence of this disease. The most important findings of the study are as follows: (i) 11.4% of the investigated serum samples of goats using RBPT had antibodies against brucellosis, (ii) the seroprevalence was higher in goats raised at farm in comparison with goats purchased from local animal markets, nevertheless, such variations were not found among the categories of other risk factors, and (iii) location, breed, herd size, and flock expansion and/or animal replacement were associated with positive brucellosis status.

In Sudan, brucellosis occurs in all farm and some wildlife animal species and humans [7,17]. Two brucella species have been isolated from clinically diagnosed cases of brucellosis in the country. These were *B. abortus* (biovars 1, 3, 6 and 7) and *B. melitensis* (biovars 2 and 3) [17]. The first isolation of *B. melitensis* was from milk samples collected in the middle of the former century from goats, sheep, and cows in central Sudan [13]. *B. melitensis* was once again isolated during the 1990s in the western part of the country from samples collected from a mixed flock of sheep and goats [11]. The seroprevalence reported in this study is identical to the ones reported in Khartoum and El-Gedarif states [18,19]. However, it is higher than many reports from different parts of the country which were between 0.3% and 6.0% [13,14,20-24]. On the other hand, it is lower than a report from the northern part of Sudan which was 16.3% [25].

In neighboring countries, Egypt and Ethiopia, similar findings were recently reported [6,26]. However, in the past 2 years, Molla and Dedil [27] found a prevalence of 0.0% in samples collected from goats in South Western Ethiopia. In the same context, in other African countries, seroprevalences varied from 0.0% to 5.8% [28-31]. In resource-limited settings in other parts of the world, seroprevalences were also low in comparison to the findings of the present study and were between 3.2% and 9.8% [4,32]. Nonetheless, the seroprevalence reported herein is lower than the ones reported in Libya and Nigeria. In these countries, the seroprevalences ranged from 14.5% to 31% [3,33]. It is also lower than the seroprevalences reported in Jordan (24.6-27.7%) [34].

The differences observed between the seroprevalence reported in this study and the seroprevalences observed in previous studies in Sudan and countries with similar socioeconomic status in Africa and other parts of the world could likely be attributed to the dissimilarities of the number of investigated goats. The probability of finding seropositive goats increases when a large number of animals is investigated [18]. In this study, categories of risk factors with a small number of investigated goats had a small number of seropositives. Moreover, different diagnostic methods for detection of brucellosis have been used in each study. This might be another important reason for the noticed variations. It is known that these diagnostic methods have different sensitivities and specificities. Primary binding assays such as fluorescence polarization assay, iELISA, and cELISA are more accurate than the conventional tests (RBPT and SAT) [35]. In general, high prevalences of brucellosis in goats would be expected when the predominant animal production system allows close contact between infected and susceptible animals. In addition, free uncontrolled movement of animals and poor management practices and sanitary measures might facilitate the transmission of brucellosis between goats [18]. All of the interviewed owners/herders claimed that their goats were not vaccinated against brucellosis. It remains unknown whether this is actually true or not as it difficult to differentiate between previously vaccinated and not vaccinated goats. However, if it is true that the investigated goats were not vaccinated, this would serve as an additional explanation for the reported seroprevalence in this study.

The pre-purchase testing policy may perhaps explain why goats purchased from local animal markets had a lower seroprevalence in comparison with goats raised in the farm. Animals arriving at local animal markets are subjected to serological testing for brucellosis. Test-positive goats are sold for meat and taken to the slaughterhouse immediately. Previous studies investigating potential risk factors that enhance the seropositivity of brucellosis among farm animals in Sudan did not observe differences in the seroprevalences of the categories of the investigated risk factors [7,18].

The correlation between location and herd size and the seropositivity of brucellosis could likely be attributed to animal population density. Aforementioned, a large number of animals could likely result in more and frequent direct and indirect contact amongst the animals, hence, transmission of infectious diseases increases. Sharq an-Nil locality has the largest animal population compared with other localities. It has the highest proportion of RBPT-positive reactors also. This was typifying the findings made elsewhere [32,34]. Moreover, the breed was found to be correlated with the seroprevalence of brucellosis in goats. This could be due to natural resistance. Indigenous goats are more resistant to brucellosis than foreign goats [36].

In this study, anti-brucella antibodies were detected in goats in Khartoum state. This suggests that the zoonotic transmission of brucella species through consumption of contaminated milk or meat of infected goats is possible. Therefore, goats’ milk and meat should be very well-boiled and cooked before consumption. In addition, inter- and intra-species (goat-to-goat and goat-to-other animal species) transmission of brucellosis from infected goats to naïve goats, sheep, cattle, and camels can also happen. Unless proper control and management measures such as vaccination and test-and-slaughter policy are efficiently and adequately applied.

Although brucellosis is expected to be more prevalent among sexually mature goats, female goats, goats with a history of abortion and retained placenta, and in pregnant goats as result of biological reasons such as female sex hormones [2], such correlations could not be established in this study. This might perhaps denote to that the clinically diagnosed abortion cases were because of causes other than brucellosis.

The strengths of this study included investigating individual animal and management potential risk factors as well as using conventional tests (RBPT and SAT) and primary binding assay (cELISA) for screening and confirming positive reactions. However, the number of investigated goats is small. This was one limitation of the study.

## Conclusion

Brucellosis was detected in goats in all surveyed localities of Khartoum state using serial testing scheme, i.e., one serological test for screening and two tests for confirming the positive results of the screening test. In addition, potential risk factors for the seroprevalence of brucellosis in goats were location, breed, herd size, and raising animals at the farm for flock expansion and/or animal replacement. Our results highlight the need for further epidemiological studies in Khartoum state to understand brucellosis in goats better. An effort should be made and focused on raising the awareness of goat owners/herders about brucellosis and educate them about the benefits of vaccination. The economic impact of brucellosis in goats in Khartoum state should be studied too.

## Authors’ Contributions

EMM, AAM, and EME participated in the design and coordination of the study; EMM collected samples and conducted laboratory work; EMM, AAM, MAA, and YAS were involved in data analysis and interpretation and manuscript reviewing and correction; YAS, HHI, and SEM drafted and reviewed the manuscript. All authors read and approved the final manuscript.
